# Development of a Flexible Artificial Lateral Line Canal System for Hydrodynamic Pressure Detection

**DOI:** 10.3390/s17061220

**Published:** 2017-05-26

**Authors:** Yonggang Jiang, Zhiqiang Ma, Jianchao Fu, Deyuan Zhang

**Affiliations:** 1 School of Mechanical Engineering and Automation, Beihang University, Beijing 100191, China; mazqbuaa@163.com (Z.M.); fujianchao1990@126.com (J.F.); zhangdy@buaa.edu.cn (D.Z.); 2 International Research Institute for Multidisciplinary Science, Beihang University, Beijing 100191, China

**Keywords:** lateral line, flexible sensor, pressure sensor, biomimetic, piezoelectric

## Abstract

Surface mounted ‘smart skin’ can enhance the situational and environmental awareness of marine vehicles, which requires flexible, reliable, and light-weight hydrodynamic pressure sensors. Inspired by the lateral line canal system in fish, we developed an artificial lateral line (ALL) canal system by integrating cantilevered flow-sensing elements in a polydimethylsiloxane (PDMS) canal. Polypropylene and polyvinylidene fluoride (PVDF) layers were laminated together to form the cantilevered flow-sensing elements. Both the ALL canal system and its superficial counterpart were characterized using a dipole vibration source. Experimental results showed that the peak frequencies of both the canal and superficial sensors were approximately 110 Hz, which was estimated to be the resonance frequency of the cantilevered flow-sensing element. The proposed ALL canal system demonstrated high-pass filtering capabilities to attenuate low-frequency stimulus and a pressure gradient detection limit of approximately 11 Pa/m at a frequency of 115 ± 1 Hz. Because of its structural flexibility and noise immunity, the proposed ALL canal system shows significant potential for underwater robotics applications.

## 1. Introduction

The lateral line is a mechanosensory organ ubiquitous in fish that supports a variety of important functions, such as predation, copulation, and schooling. It comprises a large array of distinct mechanosensory neuromasts, each of which can respond to movement of the surrounding fluid. In accordance with their morphologies, the neuromasts are divided into two categories: (1) superficial neuromasts located on the body surface of fish and (2) canal neuromasts located in the fluid-filled canals under the lateral line scales. The difference in morphology results in different neuromast functions—superficial neuromasts detect the velocity distribution and canal neuromasts detect the pressure gradients—which allow fish to construct hydrodynamic rather than visual images of their surrounding environment [1,2,3].

**Figure 1:**
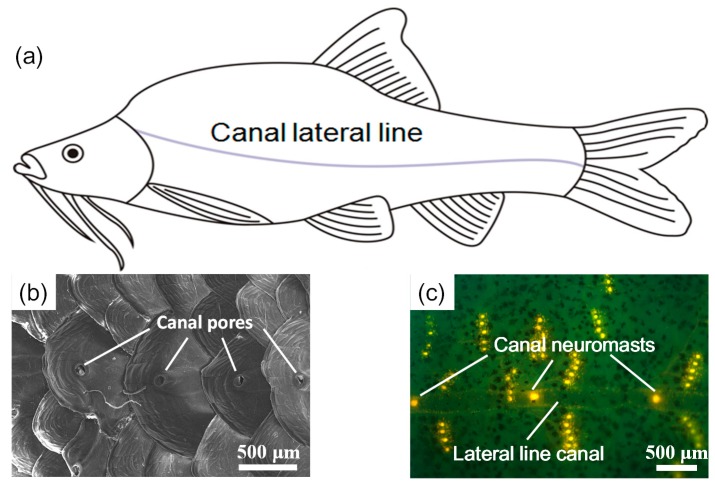
Lateral line canal system of the cavefish, *Sinocyclocheilus Macrophthalmus*: (**a**) Schematic diagram of the canal lateral line; (**b**) Scanning electron microscope image of canal pores; (**c**) Fluorescence microscope image of canal neuromasts.

Compared with surface fish, some cavefish living in dark environments possess more sensitive lateral line systems to compensate for their degenerative vision [4]. In a prior study, we investigated the lateral line systems of *Sinocyclocheilus Macrophthalmus* and *S. Microphthalmus*, which live in the karst caves in Guangxi, China [5]. As shown in Figure 1, both superficial and canal lateral line systems can be observed in *S. Macrophthalmus*. Results from behavior experiments indicated that the lateral line canal system in *S. Macrophthalmus* was highly sensitive to dipole vibrations of 60–90 Hz. The high sensitivity of the lateral line canal system is complemented by its biomechanical filtering mechanism, in which the canal-pore structure constitutes a high-pass filter and the fluid-cupula interaction results in a band-pass filter [6,7,8].

Artificial lateral line (ALL) systems are important for many engineering applications, especially for underwater robots and vehicles. Inspired by the unparalleled underwater dynamic flow and pressure sensing mechanism of the lateral line system of fish, researchers have previously developed ALL systems using various transduction mechanisms. For example, Fan et al. [9] initially proposed an artificial hair cell sensor based on a piezoresistive silicon microcantilever with a plastically bent vertical cilium. Peleshanko et al. [10] decreased the artificial hair cell threshold sensitivity for the effective flow velocity from 1 mm/s to 0.01 mm/s by fabricating a hydrogel cupula on the piezoresistive sensor. Yang et al. [11] developed an ALL canal system that integrated piezoresistive sensors into a soft polydimethylsiloxane (PDMS) canal and showed significantly improved noise immunity for hydrodynamic detection compared with superficial sensors. In various follow-on studies, Chen et al. [12] and Yang et al. [13] supplemented piezoresistive transduction designs with an array of out-of-plane hot-wire anemometers to imitate the lateral line superficial systems of fish with a detection threshold of 0.2 mm/s. More recently, Herzog et al. [14] proposed a μ-biomimetic flow sensor using an optical detection principle. A photodiode detected deflection of each PDMS-lamella on a silicon chip induced by fluid flow inside the canal. Kottapalli et al. [15] and Asadnia et al. [16] considered a piezoelectric ALL canal system that uses a lead zirconate titanate (PZT)/silicon diaphragm as the sensing structure and elucidates the ability of high-pass filtering to eliminate low-frequency noise for underwater acceleration sensing.

To date, most of the proposed sensing structures for ALL canal systems use either silicon or piezoelectric ceramics. Xu et al. [17] introduced a flexible shear-stress sensor array by using a parylene C based hinge structure to link the rigid Si sensors and CMOS circuits. Though the IC-integrated flexible shear-stress sensor array showed incomparable advantages in sensitivity and resolution, its flexibility was limited by the rigid silicon sensing elements. Piezoelectric polymers and ion-exchange polymer metal composites (IPMC) are most promising among the various flexible transduction materials. Prior studies have confirmed that IPMC can respond to static displacement and are sensitive to flow rate when used in ALL systems [18,19]. Polyvinylidene fluoride (PVDF) is a piezoelectric polymer that offers high piezoelectric voltage constants and is widely used in microelectromechanical systems [20,21]. In our proposed ALL canal system, PVDF was used in the cantilevered flow-sensing elements, and PDMS was used as the material for the canals. Therefore, the ALL canal system is fully flexible, which is important for underwater hydrodynamic detection.

## 2. Design, Fabrication, and Testing Procedures

### 2.1. Design of the ALL Canal System

**Figure 2:**
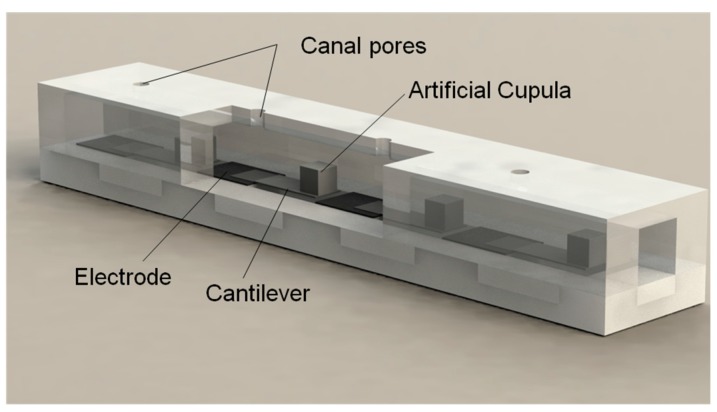
Schematic structure of the proposed ALL canal system.

Inspired by the lateral line canal systems of cavefish, Figure 2 shows the array of pressure sensors used in the proposed ALL canal system. This design comprises four pressure-sensing units, each consisting of a PVDF/polypropylene laminated cantilever with a biomimetic cupula on the cantilever tip. The PVDF serves as the electromechanical transduction material for its high piezoelectric voltage coefficient. In accordance with the morphology and function of the natural lateral line canal system, PDMS was used to fabricate the microfluidic canal. 

All cantilevered sensing elements are coupled with an internal liquid medium in a microfluidic canal. Pore-like openings in the PDMS connect the canal’s internal medium with the external medium. The pressure difference between adjacent pore-like openings causes the internal medium to flow, consequently applying pressure to the cross-sectional areas of the biomimetic cupulas. This pressure causes stress changes in the PVDF/polypropylene cantilevers, which generate piezoelectric charges on the PVDF film surface. As a result, the vibration-induced pressure variation of the external medium can be estimated using the charge output of the sensing elements.

To simplify the design calculations for the proposed ALL canal system sensor units, various assumptions were made: (1) the near-field flow was assumed local and incompressible; (2) the propagating sound wave was assumed negligible [7]; and (3) the force applied to the PDMS cupula was assumed to originate only from the internal medium flow induced pressure, *p*. The flexible PVDF/polypropylene cantilevers function at their bending mode; the bending moment can be expressed as

(1) $$M_{p}=\frac{pab^{2}}{2},$$

where, *a* and *b* are the width and height of the PDMS cupula, respectively.

**Figure 3:**
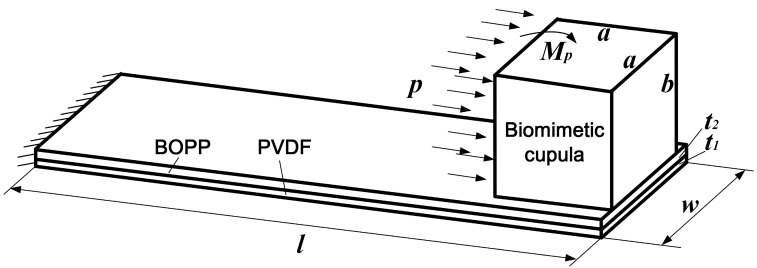
Mechanics of the cantilevered flow-sensing element in the artificial canal.

Figure 3 shows the mechanics of the cantilevered flow-sensing element in the artificial canal. In this case, the piezoelectric film’s surface charge density primarily originates from the normal stress, σ_1_, in the PVDF layer along the cantilever’s lengthwise direction. For a cantilever with a PVDF layer thickness of *t*_1_ and a polypropylene layer thickness of *t*_2_, σ_1_ can be calculated as

(2) $$\sigma_{1}=\frac{E_{1}M_{p}[z-\left(t_{1}+t_{2}\right)/2]}{E_{1}I_{1}+E_{2}I_{2}}+\frac{pabE_{1}}{w(E_{1}t_{1}+E_{2}t_{2})},$$

where, *E*_1_ and *E*_2_ are the Young’s moduli of the PVDF and polypropylene, respectively; *I*_1_ and *I*_2_ are the moments of inertia around the neutral axis for the PVDF and polypropylene, respectively; *z* is the distance from the top surface of the PVDF layer; *w* is the width of the cantilever. The thickness of the electrodes on each side of the PVDF film is neglected.

Given a cantilever length, *l*, and width, *w*, the surface charge quantity, *Q*, can be derived by integrating the charge density in the PVDF layer as

(3) $$Q=\int_{0}^{l}\int_{0}^{w}\int_{t_{2}}^{t_{1}+t_{2}}d_{31}(\sigma_{1}/t_{1})dzdxdy,$$

where *d*_31_ is the piezoelectric strain coefficient of the PVDF film. The sensitivity of each sensor unit can be expressed as

(4) $$Q_{p}=Q/p,$$

**Figure 4:**
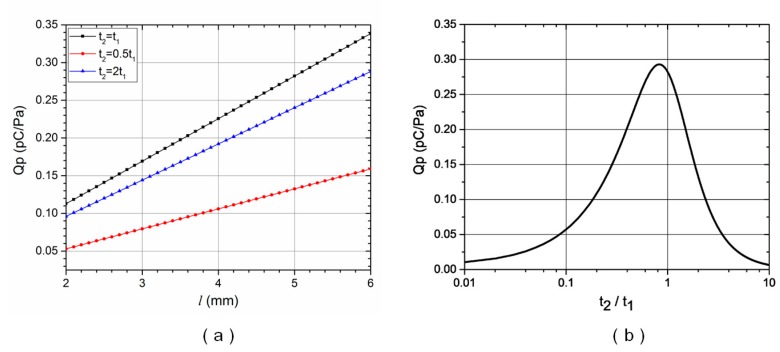
Sensor sensitivity: (**a**) As a function of cantilever length and polypropylene layer thickness; (**b**) As a function of the polypropylene-to-PVDF layer thickness ratio for a cantilever length of 5 mm.

For a biomimetic cupula with a cubic side length of *a* = *b* = 1.5 mm, a cantilever width of *w* = 2 mm, and a PVDF film thickness of *t*_1_ = 45 μm, the sensitivity of each sensor unit, *Q*_p_, can be calculated for various cantilever lengths, *l*, and polypropylene layer thicknesses, *t*_2_, using Equations (1)–(4). Figure 4a shows that sensor sensitivity, *Q*_p_, consistently increased as cantilever length, *l*, increased, but the rate of increase varied depending on the polypropylene-to-PVDF layer thickness ratio, *t*_2_/*t*_1_. Figure 4b shows the relationship between *Q*_p_ and *t*_2_/*t*_1_. For a cantilever length of *l* = 5 mm, sensor sensitivity was maximized when *t*_2_/*t*_1_ was approximately 0.8. In this calculation, the PVDF and polypropylene Young’s moduli were *E*_1_ = 2.5 and *E*_2_ = 2.2 GPa, respectively, and the piezoelectric strain coefficient of the PVDF film was *d*_31_ = 21 pC/N.

Based on above calculation, we used a PVDF layer thickness of *t*_1_ = 45 μm and a polypropylene layer thickness of *t*_2_ = 41 μm for the laminated cantilevered sensors. To support technological accessibility during system fabrication and packaging, the PVDF/polypropylene cantilever was designed with a length of *l* = 5 mm and a width of *w* = 2 mm. The PDMS cupula located on the cantilever tip was designed with a cubic side length of *a* = *b* = 1.5 mm. The cross-sectional dimension of the PDMS canal was 3 × 3 mm and the distance between adjacent sensor units was 10 mm. Four 1 mm diameter pores spaced 10 mm apart were included along the PDMS microfluidic canal to transfer the pressure differences between the external medium and internal flow.

### 2.2. Fabrication of the ALL Canal System

**Figure 5:**
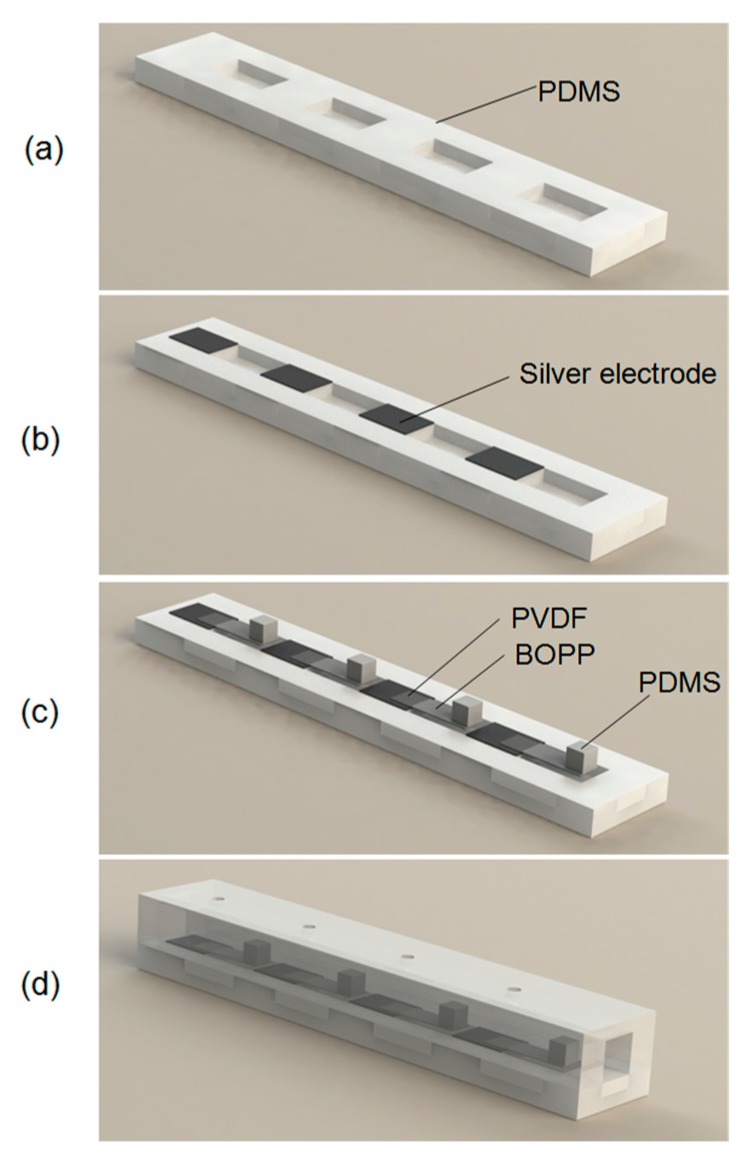
Schematic illustration of stepwise fabrication process: (**a**) Lower PDMS canal structure cast; (**b**) Electrode deposition on lower PDMS canal; (**c**) PVDF/polypropylene cantilever and PDMS cupula assembly; (**d**) Upper canal-pore structure cast and lower-upper PDMS canal structure bond.

Figure 5 illustrates the stepwise fabrication process used in this study. First, the PDMS microfluidic canal structure—comprising lower rectangular cavities to support cantilever bending and upper canals with pore-like openings—was fabricated by PDMS molding process (Figure 5a). Negative molds of the required canal structures were machined with aluminum alloy. A liquid PDMS elastomer and a curing agent were mixed using a 10:1 ratio by weight. This degassed mixture was then poured into molds and cured at 70 °C for 4 h in a drying cabinet. The PDMS structures were then peeled out of the molds. Next, electrodes were formed on the lower PDMS canal by selectively depositing a thin layer of silver with a stencil mask. A sputtering system (JPGF-450D, Beijing Beiyi Innovation Vacuum Technology Co., LTD, Beijing, China) was used with RF power of 100 W and processing time of 10 min (Figure 5b). A PVDF sheet sandwiched by 300 nm thick aluminum layers (upper and lower electrodes of the PVDF sheet) was laminated to the polypropylene layer with acrylate adhesive. The PVDF/polypropylene sheet was cut into designed cantilever structures, which formed the flow-sensing elements. 

**Figure 6:**
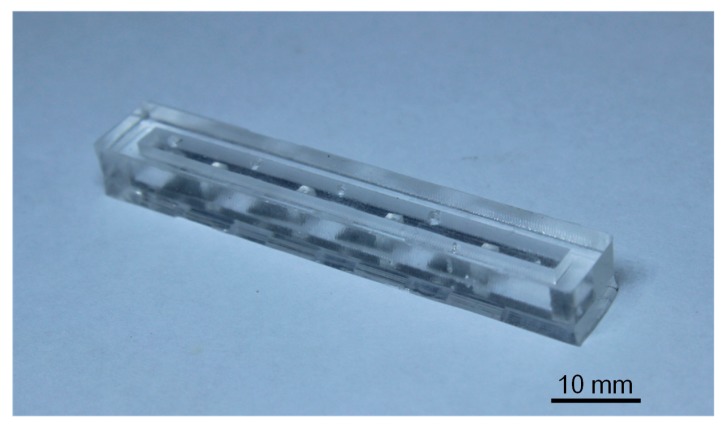
Photographic image of the final ALL canal system prototype.

The PDMS cupula was mounted on the apex of the PVDF/polypropylene cantilever. Conductive silver paste was used to electrically connect the lower electrodes on the PVDF layer with the electrodes on the PDMS canal (Figure 5c). The electrodes of the PVDF/polypropylene cantilever were connected to a fine coaxial cable using adhesive silver paste. The signal line and shielding layer in the coaxial cable were connected to upper and bottom sliver electrodes, respectively. Cantilevered flow sensors were uniformly encapsulated with a 2 μm thick parylene layer using a chemical vapor deposition system (PDS-2010, Specialty Coating System, Indianapolis, IN, USA) to provide waterproofing and electrical insulation. Finally, the lower and upper PDMS canal structures were adhesively bonded together to form the final ALL canal system (Figure 5d). Figure 6 provides a photographic image of the final ALL canal system prototype with four cantilevered flow-sensing elements.

### 2.3. Testing of the ALL Canal System

**Figure 7:**
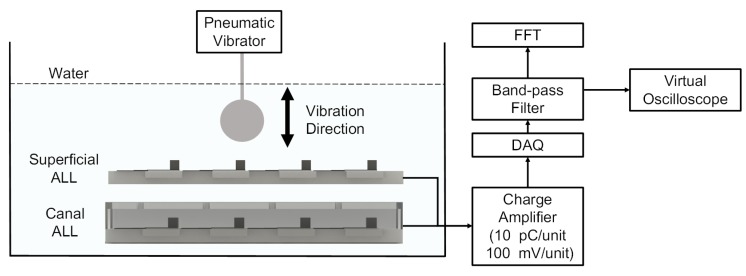
Schematic diagram of the experimental setup.

Both the ALL canal system prototype and its superficial counterpart were characterized in a series of experiments performed in a 110 × 40 × 30 cm water tank using a dipole vibration source. Figure 7 shows the experimental setup. The ALL canal system prototype was horizontal in the water with the dipole source (comprising a 10-mm diameter sphere attached to a pneumatic vibrator) above the system’s cupula.

The signal outputs from the ALL canal system prototype’s sensing elements were amplified by a charge amplifier (NEXUS Conditioning Amplifier-2692, Brüel & Kjær, Nærum, Denmark) with a transducer sensitivity of 10 pC/unit and output sensitivity of 100 mV/unit. This data was then collected by a USB Data Acquisition System (USB-4761, Advantech, Taipei, Taiwan) with a data-sampling rate of 2000 Hz. After passing through a virtual band-pass filter with a low cutoff frequency of 5 Hz and a high cutoff frequency of 150 Hz, the voltage output wass converted into the frequency domain using a Fast Fourier Transformation (FFT). The amplitude of the voltage output was simultaneously measured. The signal outputs expressed in the frequency and time domains were depicted graphically. 

In each of the experiments, the dipole vibration frequency could be adjusted manually, but the amplitude (which is independent of frequency) was fixed at 0.3 mm. The vibration direction was perpendicular to the top surface of the ALL canal system prototype. The dipole source was initially placed at a vertical distance of 35 mm above the system’s cupula (measured from the sphere’s center to the top surface of the cupula) and vibration frequencies were varied with randomized values between 5–150 Hz). In subsequent experiments, the vibration frequency was held constant at 115 ± 1 Hz while the vertical distance of the dipole source varied from 10 to 60 mm.

## 3. Results and Discussion

**Figure 8:**
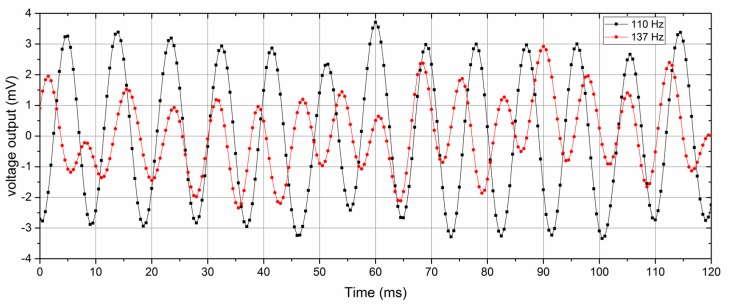
ALL canal system voltage output at vibration frequencies of 110 and 137 Hz.

As the dipole source maintained a constant amplitude of 0.3 mm, the amplitude variation in the output voltage resulting from the vibration frequency represents the frequency response of the ALL canal system prototype. Under the same experimental conditions (dipole vibration frequency and relative position of ALL device and dipole source), it found that the sensing elements performed with a good uniformity. Figure 8 shows the voltage output of the ALL canal sensor at vibration frequencies of 110 and 137 Hz. The noise level of the sensor output was approximately 2 mV.

**Figure 9:**
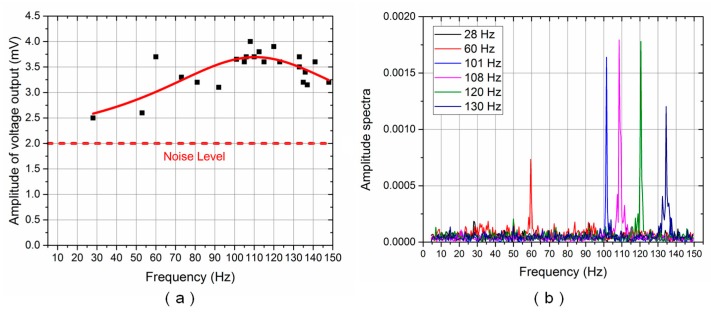
ALL superficial system frequency response for random vibration frequencies: (**a**) Voltage output amplitude (solid red line reflects Lorentz fit); (**b**) Amplitude spectra.

**Figure 10:**
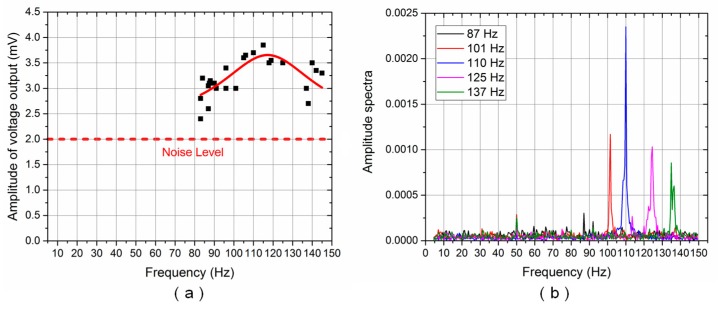
ALL canal system frequency response for random vibration frequencies: (**a**) Voltage output amplitude (solid red line reflects Lorentz fit); (**b**) Amplitude spectra.

Figure 9a and Figure 10a show the frequency response of the ALL superficial and canal sensors, respectively. The Lorentz fit [22] was also conducted to determine the peak frequency response of the ALL canal system prototype. The solid red line reflects the results of the Lorentz fit. The amplitude of the voltage output reached its maximum value at approximately 110 Hz for both the ALL superficial and canal sensors.

Figure 9b and Figure 10b show the amplitude spectra of the voltage output for the ALL superficial and canal sensors, respectively, using various random vibration frequency values between 5–150 Hz. The Lorentz fit and amplitude spectra indicated that the resonance frequency of the cantilevered flow-sensing element was approximately 110 Hz. The theoretical resonance frequency of the cantilever sensor in air was calculated as approximately 206 Hz. The resonance frequency decreased while the ALL device moved from air into water, which probably originated from the effect of high density and viscous characteristic in water [23]. The lowest frequency stimulus detected by the ALL canal sensors was approximately 84 Hz. It suggests that the high-pass filtering function in the artificial canal, which attenuates low-frequency stimulus, was effective despite the high noise level of the prototype in the time domain.

**Figure 11:**
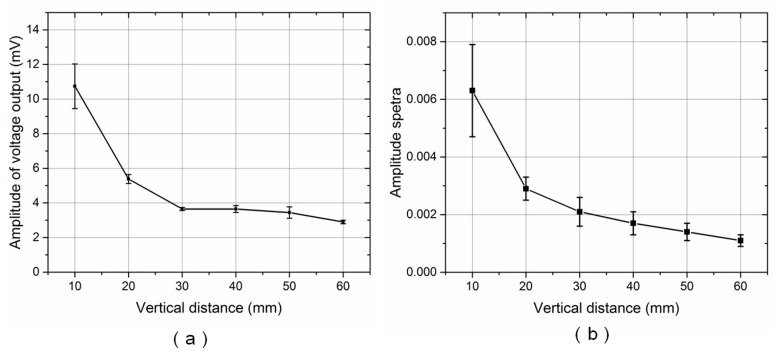
ALL canal system response for various dipole source vertical distances and a vibration frequency of 115 ± 1 Hz: (**a**) Voltage output amplitude; (**b**) Amplitude spectra.

In subsequent experiments, the vibration frequency was fixed at 115 ± 1 Hz while the vertical distance between the dipole source and the ALL canal system’s cupula increased from 10 to 60 mm. Figure 11a,b show the voltage output amplitude and amplitude spectra, respectively. In terms of overall trend, both the voltage output amplitude and amplitude spectra decreased as the vertical distance to the dipole source increased. The voltage output is almost immersed in noise, though the average amplitude spectra is 0.0011 at a distance of 60 mm. 

Using a dipole analytical model [11,24], the pressure gradient between the pore, *P*_1_, and the adjacent pore, *P*_2_—with a spacing of *Δx* = 10 mm—can be calculated as

(8) dpdx=(1l02−l0(l02+(Δx)2)32)ρrs32ΔxAssin(2πft),

where, *f* is the vibration frequency of the sphere, *A*_s_ is its acceleration amplitude, and *r*_s_ is its radius. For a vibration frequency of *f* = 115 ± 1 Hz and a vertical distance to the sphere of *l*_0_ = 60 mm, the pressure gradient between *P*_1_ and *P*_2_ was calculated as 11 Pa/m. Because the spacing between two adjacent canal pores is 10 mm, this finding suggests that the proposed ALL canal system can effectively detect a pressure difference of 0.11 Pa between the adjacent canal pores. Fernandez et al. [25] proposed a lateral line-inspired sensor system using a high-density array of piezoresistive pressure sensors with a pressure resolution of 1.0 Pa. Yaul et al. [26] presented a flexible ALL pressure sensor array using carbon black as the strain gauge, achieving a pressure resolution of 1.5 Pa. Kottapalli et al. [27] developed an lateral-line inspired sensors based on liquid crystal polymer, which turned out to be 19% more sensitive than silicon strain gauges. Therefore, the ALL canal system in our study is much more sensitive than the reported devices at its resonance frequency. However, the artificial lateral line canal device was unable to detect static signals, as piezoelectric transduction mechanical was used. 

## 4. Conclusions

The lateral line canal systems of cavefish, such as *Sinocyclocheilus Macrophthalmus*, show extremely high sensitivities to proximate vibrational disturbances. In this study, we developed an artificial lateral line canal system that serves as a flexible pressure sensor array. A simple fabrication process was established, which uses PVDF/polypropylene laminated cantilevers as flow-sensing elements and PDMS for the artificial canals. Experimental results indicated that the proposed canal structure functioned effectively as a high-pass filter to attenuate low-frequency stimulus. The proposed ALL canal system was able to detect a pressure gradient as low as 11 Pa/m at a frequency of 115 ± 1 Hz. The performance of this bioinspired system can be further improved by modifying the canal geometry and optimizing the sensing structures. Future work will also focus on the miniaturization of the system using batch fabrication processes.
